# A Sensitive Electrochemical Sensor Based on Sonogel-Carbon Material Enriched with Gold Nanoparticles for Melatonin Determination

**DOI:** 10.3390/s22010120

**Published:** 2021-12-24

**Authors:** Cecilia Lete, David López-Iglesias, Juan José García-Guzmán, Sorina-Alexandra Leau, Adina Elena Stanciu, Mariana Marin, José Maria Palacios-Santander, Stelian Lupu, Laura Cubillana-Aguilera

**Affiliations:** 1 Electrochemistry-Corrosion Department, Institute of Physical Chemistry “Ilie Murgulescu” of the Romanian Academy, 202 Splaiul Independentei, 022328 Bucharest, Romania; sorina.leau@stud.fim.upb.ro (S.-A.L.); mmaria@icf.ro (M.M.); 2 Department of Analytical Chemistry, Faculty of Sciences, Campus de Excelencia Internacional del Mar (CEIMAR), Institute of Research on Electron Microscopy and Materials (IMEYMAT), University of Cadiz, República Saharaui, S/N. Puerto Real, 11510 Cadiz, Spain; david.lopeziglesias@uca.es (D.L.-I.); juan.garcia@inibica.es (J.J.G.-G.); josem.palacios@uca.es (J.M.P.-S.); laura.cubillana@uca.es (L.C.-A.); 3 Department of Analytical Chemistry and Environmental Engineering, Faculty of Chemical Engineering and Biotechnologies, University Politehnica of Bucharest, 1-7 Polizu Gh. Street, 011061 Bucharest, Romania; 4 Department of Carcinogenesis and Molecular Biology, Institute of Oncology Bucharest, 252 Fundeni, 022328 Bucharest, Romania; adina2706@gmail.com

**Keywords:** Sonogel-Carbon electrode material, Au nanoparticles, poly(3,4-ethylenedioxythiophene), electrochemical sensor, melatonin

## Abstract

In this work, the development of an electrochemical sensor for melatonin determination is presented. The sensor was based on Sonogel-Carbon electrode material (SNGCE) and Au nanoparticles (AuNPs). The low-cost and environmentally friendly SNGCE material was prepared by the ultrasound-assisted sonogel method. AuNPs were prepared by a chemical route and narrow size distribution was obtained. The electrochemical characterization of the SNGCE/AuNP sensor was carried out by cyclic voltammetry in the presence of a redox probe. The analytical performance of the SNGCE/AuNP sensor in terms of linear response range, repeatability, selectivity, and limit of detection was investigated. The optimized SNGCE/AuNP sensor displayed a low detection limit of 8.4 nM melatonin in synthetic samples assessed by means of the amperometry technique. The potential use of the proposed sensor in real sample analysis and the anti-matrix capability were assessed by a recovery study of melatonin detection in human peripheral blood serum with good accuracy.

## 1. Introduction

Melatonin (N-acetyl-5-methoxytryptamine, MEL) belongs to the organic compound class known as 3-alkylindoles. This compound is an endogenous lipophilic hormone secreted by the pineal gland in mammalian brain. Due to its antioxidant capacity, MEL helps to regulate the sleep–wake cycle and the production of other hormones [1,2,3]. It has been used in the treatment of several gastrointestinal diseases such as irritable bowel system and ulcerative colitis as well as in neurodegenerative diseases such as Alzheimer’s and Parkinson’s [4]. MEL is also recognized for its influence in the aging process, sleep efficiency, seasonal affective disorder, retinal physiology, mood regulation, dreaming, pubertal development in some species, and modulates blood pressure [5,6]. Furthermore, MEL is a critical free radical scavenger and upregulates the immune response [7]. Due to its important roles in numerous pathological, physiological, and biological processes, unsuitable levels of melatonin in our bodies can lead to different diseases. For this reason, the development of a sensitive and selective method for MEL determination is highly worthwhile for biomedical analysis and diagnosis.

Several analytical methodologies have been reported in the literature for melatonin quantitation such as high-performance liquid chromatography [8,9,10], chemiluminescence [11], spectrofluorimetry [12,13], and enzyme-linked immunosorbent assays [14]. These methods provide accurate and reliable results, but these strategies are complex and require high cost equipment and trained personnel. On the other hand, electrochemical methods have attracted great attention due to their simplicity, low cost, reliability, and fast analytical response [15]. Electrochemical methods have been exploited in the development of various electrochemical sensors for the determination of melatonin [16]. The electrochemical sensors based on carbon electrode materials have attracted great interest due to their low cost, sensitivity, chemical inertness, and easy modification. Consequently, carbon paste electrodes [17,18], waterproof paper [19], reduced graphene oxide [20], carbon nanofibers embedded with FeCo alloy nanoparticles [21], and glassy carbon modified with several compounds such as carbon black dispersion [22], palladium nanoparticles [23], and multiwalled carbon nanotube-dihexadecyl hydrogen phosphate film [24], among others, have been investigated.

As a cost effective and environmentally friendly alternative, Sonogel-Carbon electrodes have been obtained by a sonocatalysis method based on the sol–gel route. In this way, ultrasonic cavitation is achieved, promoting hydrolysis and polycondensation steps in the absence of any kind of organic solvent. The Sonogel-Carbon based material has been used in sensing and biosensing purposes due to its outstanding properties such as wide operational potential window, low residual current, good mechanical properties, and good stability in different solvents. In addition, it is easily modified by the incorporation of several compounds such as enzymes [25,26,27] and metallic nanoparticles [28,29].

In this work, Sonogel-Carbon (SNGCE) and Sonogel-Carbon-modified with gold nanoparticles (SNGCE/AuNPs) are proposed as sensing elements of an electrochemical sensor for melatonin determination. The affordable cost and eco-friendly preparation of the SNGCE material support its investigation as a sensing element in the development of an electrochemical sensor for melatonin. The tailoring and design of the proposed electrochemical sensor was achieved by exploring the well-known properties of gold nanoparticles for electrochemical sensing applications [30,31]. The prepared sensing materials were further investigated in terms of their electrochemical and electroanalytical properties and performance toward melatonin determination. The morphology and the optical properties of the prepared materials were studied by dynamic light scattering and UV–Vis spectroscopy. The sensing materials were applied in the determination of melatonin in synthetic samples. The analytical performance of the proposed sensor was assessed, and its potential applicability was demonstrated by recovery studies in real samples such as human peripheral blood serum.

## 2. Materials and Methods

### 2.1. Reagents and Solutions

Methyltrimethoxysilane (MTMOS) was obtained from Merck (Darmstadt, Germany) and hydrochloric acid from Panreac (Barcelona, Spain). Sodium citrate trihydrate was purchased from Scharlau (Scharlab, Barcelona, Spain) and potassium tetrachloroaurate(III) and melatonine from Sigma–Aldrich (Sigma, Steinheim, Germany). Graphite powder natural, high purity −200 mesh, 99.9999% (metal basis), was from Alfa-Aesar (Johnson Matthey GmbH, Sulzbach, Germany). Sodium acetate (Sigma-Aldrich, Steinheim, Germany), glacial acetic acid (Sigma-Aldrich, Steinheim, Germany), potassium hexacyanoferrate(III) (Sigma-Aldrich, Steinheim, Germany), potassium hexacyanoferrate(II) trihydrate (Sigma-Aldrich, Steinheim, Germany), monobasic potassium phosphate (Sigma-Aldrich, Steinheim, Germany), and dibasic potassium phosphate (Sigma-Aldrich, Steinheim, Germany) were of analytical grade. Standard melatonin was purchased from Sigma-Aldrich. Stock solution of melatonin with concentrations of 1.0 × 10^−2^ M for cyclic voltammetry measurements and 1.0 × 10^−4^ M for analytical applications studies, respectively, were prepared in ethanol due to its low solubility in water. Double distilled water was used for the preparation of all aqueous solutions. Glass capillary tubes, i.d. 1.15 mm, were used as the bodies of the sonogel composite electrodes. Nitrogen N-55 type was used to obtain inert atmospheres and deaerating solutions in the measuring cell. All reagents were of analytical grade and used as received without further purification.

### 2.2. Instrumentation

The synthesis of the SNGCE material as well as the ultrasonic synthesis of AuNPs were carried out sonicating with a high-power ultrasound generator, SONICATOR 3000, from MISONIX (MISONIX, Inc., Farmingdale, NY, USA) equipped with a 13-mm titanium tip, which provides a maximum power of 600 W. UV–Visible spectra were recorded using a Jasco V-550 (Easton, MD, USA) UV–Visible spectrophotometer.

The electrochemical measurements were performed using a three-electrode electrochemical cell connected to an Autolab potentiotstat/galvanostat 302 N (Ecochemie, The Netherlands). A glassy carbon rod (Metrohm), a Ag/AgCl/KCl (3 M) electrode (Metrohm), and an unmodified/modified SNGCE electrode were used as the auxiliary, reference, and working electrodes, respectively. The pX1000-pH module of the potentiostat was used for pH measurements. This module allows for recording of the temperature during the experiments through a combined pH/Pt1000 sensor. The temperature measurement allows for automatic pH corrections.

### 2.3. Preparation of Working Electrodes and Sensing Materials

#### 2.3.1. Sonogel-Carbon Synthesis

**Scheme 1:**
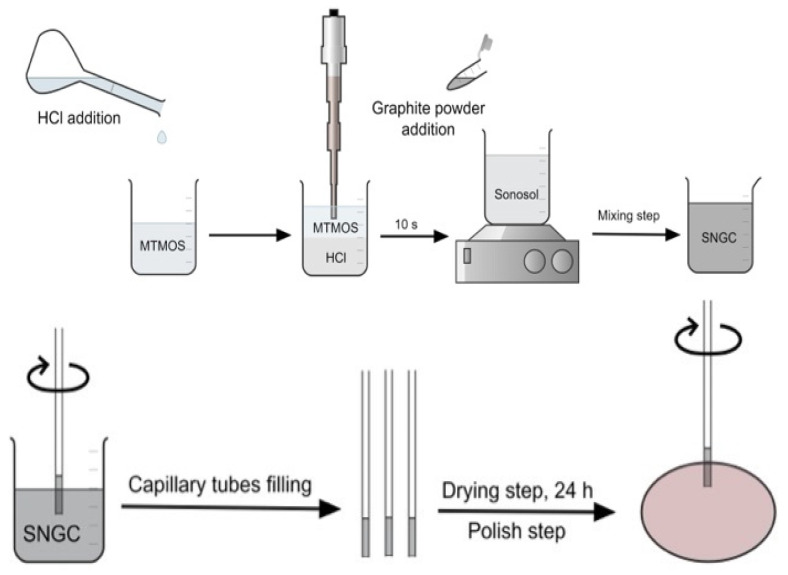
SNGCE manufacturing procedure.

**Scheme 2:**
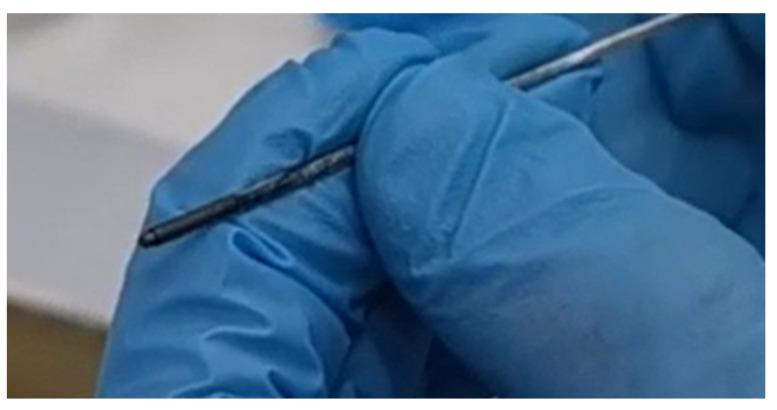
Photo image of SNCGE.

A precursor mixture containing 500 µL MTMOS and 100 µL 0.2 M HCl solutions was sonicated for 10 s, providing 10 W total power. After that, 500 mg of graphite powder was added and homogeneously dispersed into the sonosol. Capillary tubes were used as the bodies of the electrodes. After 24 h, the electrodes were finally polished, being ready to use by inserting a copper wire as the electrical contact (see Scheme 1 and Scheme 2) [32].

#### 2.3.2. Preparation of Gold Nanoparticle Solution (AuNPs)

A total of 1.25 mL of 1.5 mM potassium tetrachloroaurate solution was sonicated for 90 s. Afterward, 250 µL of 38.8 mM sodium citrate was added and the mixture was sonicated for 4 min until the formation of red wine like solution. The total time of the synthesis was 5 min and 30 s according to the literature [33].

Nanoparticle size was measured by using dynamic light scattering (DLS). UV–Vis spectrophotometry was used in order to confirm the formation of gold nanoparticles. A band located at 520 nm confirmed the formation of gold nanoparticles. Moreover, the mean diameter of the AuNPs was measured and a value of a 12.3 (±3.9) nm obtained.

#### 2.3.3. Sonogel-Carbon Modified with Gold Nanoparticles

The Sonogel-Carbon surface was modified by the drop casting of 4.1 µL gold nanoparticle solution on the electrode surface and kept in the dark at room temperature for at least 24 h. Afterward, the modified electrodes were stored at 4 °C in the absence of light. The obtained electrodes are referred to as SNGCE/AuNPs. In order to assess the deposition of AuNPs, the prepared SNGCE/AuNP was characterized in cyclic voltammetry (CV) using a ferrocyanide/ferricyanide redox couple.

### 2.4. Electrochemical Tests and Analytical Applications

The electrochemical features of SNGCE and SNGCE/AuNPs were explored by cyclic voltammetry (CV) using the redox probe potassium hexacyanoferrate(III) [K_3_Fe(CN)_6_]. The electrochemical impedance spectroscopy (EIS) measurements were performed in 0.5 M KNO_3_ solution containing 5 mM K_3_Fe(CN)_6_/K_4_Fe(CN)_6_ as the redox probe at a bias potential equal to the open circuit potential. The OCP value was +0.24 V vs. Ag/AgCl/KCl (3 M) reference electrode. The EIS spectra were recorded over the frequency range from 100 kHz to 0.05 Hz using an excitation sin wave with amplitude of 5 mV (rms) superimposed on the OCP. Following the first characterization step, CV and chronoamperometry (CA) techniques were used in the investigation of the voltammetric and amperometric responses of melatonin at the SNGCE and SNGCE/AuNP. Subsequently, the optimization of the analytical performance of the prepared sensors toward melatonin determination was performed by assessing the optimum working detection potential and pH values of the solutions. Based on the optimal experimental conditions, the analytical calibration curves for melatonin were built according to the multiple standard addition protocol by successive additions of aliquots of melatonin stock solutions in the electrochemical cell containing a supporting electrolyte solution (0.1 M PBS of pH 7). Analytical parameters such as linear response range, repeatability, sensitivity, selectivity, and limit of detection for melatonin electrochemical sensing were evaluated. The application of the proposed sensor in human peripheral blood serum for melatonin determination was performed in order to assess its potential applicability in a real sample analysis. The tests were carried out using the standard addition protocol by spiking the serum sample with a known amount of melatonin and recording the chronoamperometric response of the sensor. The recovery values were assessed from the measured current signals.

### 2.5. Blood Sample Collection and Processing

Fasting blood specimens were collected by venipuncture into BD Vacutainer SSTTM II Advance tubes with a clot activator. Serum samples were obtained by blood clotting for 30 min at room temperature and centrifugation at 1000× *g* for 15 min at 4 °C. Lipemic, icteric, or hemolytic specimens were excluded from this study. Serum samples were immediately aliquoted into labeled cryo-vials and stored at −70 °C for further analyses. The study was conducted following the principles outlined in the Declaration of Helsinki. The blood specimens were collected according to protocol no. 15140/10.09.2019 approved by the Institute of Oncology Bucharest Medical Ethics Committee. Signed informed consent was obtained from all patients.

## 3. Results

### 3.1. Electrochemical Characterization of Sensing Materials

**Figure 1:**
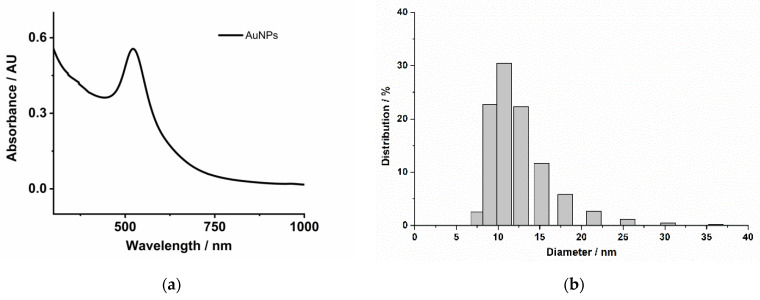
(**a**) UV–Vis spectrum of the gold nanoparticle solution. (**b**) Size distribution of the gold nanoparticles.

The modification of SNGCE with Au nanoparticles (AuNPs) was systematically investigated. The properties of the synthesized AuNPs were investigated by DLS and UV–Vis spectroscopy. The size of the AuNPs is a key point for their applications because it might influence the electrical, chemical, biological, and optical properties. All Au nanoparticle solutions were investigated in the wavelength range from 350 to 750 nm, with a resolution of 1 nm. In Figure 1a, a well-defined band at 530 nm can be observed, which is a conventional plasmon band for spherical nanoparticles. The average size of AuNPs was 12.3 (±3.9) nm and the distribution of the AuNPs’ size is displayed in Figure 1b. The small size of AuNPs could be beneficial for the development of a sensitive electrochemical sensor by an increase in catalytic sites brought by the nanoparticles dispersed onto the electrode surface by drop casting.

**Figure 2:**
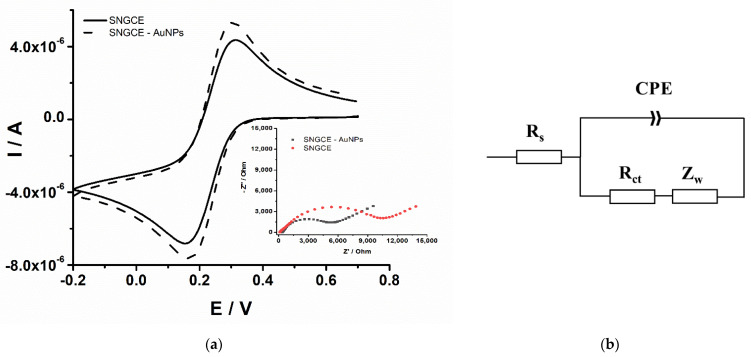
(**a**) CVs recorded at SNGCE and SNGCE/AuNPs in the presence of 5 mM K_3_Fe(CN)_6_ in 0.5 M KNO_3_ aqueous solution. Potential scan rate: 0.5 V s^−1^. Inset: EIS recorded at SNGCE and SNGCE/AuNPs in the presence of 5 mM K_3_Fe(CN)_6_/K_4_Fe(CN)_6_ in 0.5 M KNO_3_ aqueous solution. (**b**) Equivalent electrical circuit used in the fitting of the EIS data.

The prepared SNGCE/AuNPs was also characterized by CV in 0.5 M KNO_3_ solution containing 5 mM K_4_Fe(CN)_6_ as a redox probe. Figure 2a depicts the CV traces recorded at both SNGCE and SNGCE/AuNPs. There was an increase by ca. 24% in both anodic and cathodic peak currents for SNGCE/AuNPs compared to unmodified SNGCE, attesting to the good electrocatalytic properties and increased electron transfer capability of the AuNPs. This behavior was confirmed by the EIS measurements. The inset of Figure 2a exhibited a lowered R_ct_ value when the electrode was covered with AuNPs. This finding was attributed to the presence of AuNPs onto the SNGCE surface, which increased the electron transfer capability toward the redox probe.

The EIS data were fitted using an equivalent electrical circuit (EEC). The elements of the EEC were as follows (see Figure 2b): the solution resistance, (*R_s_*); the charge transfer resistance (*R_ct_*); the infinite-length Warburg diffusion impedance, (*Z_W_*); and the constant phase element (CPE, Q). The following fitted values were obtained: *R_s_* = 130.3 Ω, *R_ct_* = 10,450.0 Ω, *Q/Y_0_* = 2.8 × 10^−6^ Ω^−1^ s^-n^, *n* = 0.78, and *Z_W_* = 2.9 × 10^−3^ Ω, for SNGCE; *R_s_* = 354.9 Ω, *R_ct_* = 4750.0 Ω, *Q/Y_0_* = 6.5 × 10^−6^ Ω^−1^ s^-n^, *n* = 0.75, and *Z_W_* = 1.5 × 10^−4^ Ω, for SNGCE/AuNPs, respectively.

The Nyquist plots for both electrodes exhibited a semicircular part at high frequencies corresponding to the electron transfer process and a linear part corresponding to the diffusion at low frequencies. At high frequencies, the diameter of the semicircle represents the charge transfer resistance of the electrode interface. The SNGCE/AuNPs had a charge transfer resistance of 4750 Ω, which was lower than SNGCE (i.e., 10,450 Ω), indicating that AuNPs increased the electrical conductivity and the electron transfer capability. On the lower frequency domain, the straight line indicates that the electron transfer process on both electrodes of the redox probe is mainly controlled by diffusion.

### 3.2. Analytical Applications of Sensing Materials

#### 3.2.1. Electrochemical Behavior of Melatonin

Considering the literature data devoted to the melatonin electrochemical determination, SNGCE has never been used for the manufacture of an electrochemical sensor.

**Figure 3:**
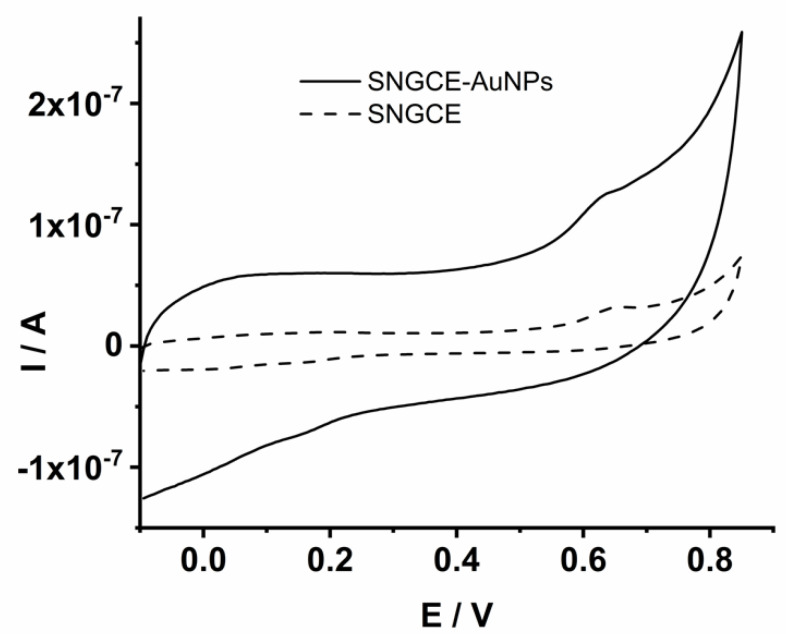
CVs recorded at SNGCE and SNGCE/AuNPs in 0.1 M PBS of pH 7 containing 20 μM MEL. Potential scan rate: 0.5 V s^−1^.

The electrochemical behavior of melatonin was investigated at the SNGCE and SNGCE/AuNPs in aqueous buffered solution. Figure 3 depicts the CVs recorded in 0.1 M PBS of pH 7 containing 20 μM MEL. The electrochemical oxidation of melatonin is an irreversible process and occurs at a potential value of 0.60 V at the SNGCE/AuNPs, which was less positive by 50 mV than the anodic oxidation peak observed at the SNGCE. It is worth noting that the peak current for SNGCE/AuNPs increased for almost four times compared with SNGCE demonstrating the advantages of AuNPs in the sensor’s construction.

**Figure 4:**
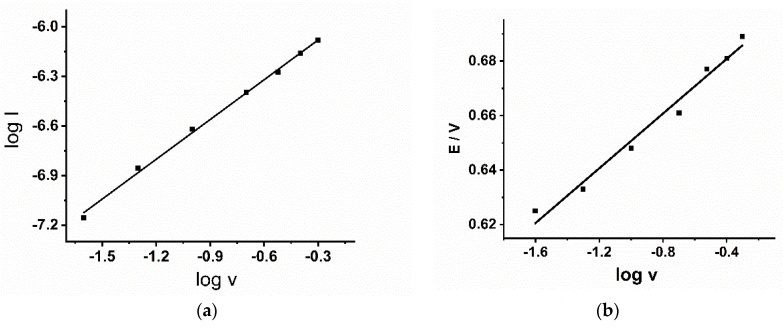
(**a**) Linear dependence of the logarithm of the anodic peak current on the logarithm of the potential scan rate for SNGCE/AuNPs. (**b**) Linear dependence of the peak potential on the logarithm of the potential scan rate for SNGCE/AuNPs.

For a proper understanding of the melatonin oxidation mechanism, the dependence of the peak current versus the potential scan rate was analyzed. The effect of the potential scan rate on the analytical response was studied in the range 25–500 mV s^−1^ in the presence of 20 µM melatonin. A linear dependence was obtained, suggesting a surface-controlled oxidation process. Moreover, the dependence of the logarithm of the peak current on the logarithm of the potential scan rate gives a slope value of 0.80 (close to 1), pointing out that the melatonin oxidation is a surface-controlled irreversible electrochemical oxidation process (Figure 4a). This dependence is described in the following equation:

Log (I_p_/A) = 0.80 Log (v/V s^−1^) − 5.8; r = 0.9983, (1)

For an irreversible electrode process, the dependence of the peak potential (*E_p_*) on the scan rate (ν) is described by the Laviron equation [34]:

E_p_ = E^0^ + (2.3 RT/αnF) log (RTk^0^/αnF) + (2.3 RT/αnF) log (ν) (2)

Laviron’s equation describes a linear relationship between the oxidation/reduction peak potential and the logarithm of the scan rate, which is valid for an irreversible adsorption process, where *E_p_* is the oxidation peak potential, *E*⁰ is the formal potential, *n* is the electron number involved in the oxidation/reduction process, *K*^0^ is the electrochemical rate constant, *α* is the transfer coefficient, *T* is the temperature (298 K), *R* is the universal gas constant (8.314 J mol^−1^ K^−1^), and *F* is the Faraday constant (96,485 C mol^−1^). Generally, *α* is assumed to be 0.5 in totally irreversible electrode processes. The number of electrons was calculated as 2.23 (*n* ≈ 2) from the slope of the *E_p_* versus log ν plot (see Figure 4b). The obtained result agrees with the oxidation mechanism proposed in the literature [24,35,36].

Since the AuNPs proved to enhance the electron transfer capability toward a model redox probe and increased electrocatalytic activity toward melatonin compared to unmodified SNGCE, the sensor based on the SNGCE/AuNPs configuration was systematically investigated to assess the analytical figures of merit of the analytical performance. Consequently, the optimization of the experimental parameters, the assessment of the analytical performance, and the analytical applications were further focused on the SNGCE/AuNPs sensor configuration.

#### 3.2.2. Optimization of Experimental Parameters

Amperometry was used in the melatonin determination due to its high analytical sensitivity. The optimization of the experimental parameters was performed in order to achieve the best electroanalytical response, and hence, the best analytical sensitivity. The chronoamperograms (CAs) were recorded in the presence of melatonin at various concentrations ranging from 0.02 to 20.00 µM. The optimization of the experimental parameters was carried out at a concentration of melatonin of 10 µM, as described below.

##### Effect of pH Supporting Electrolyte

**Scheme 3:**
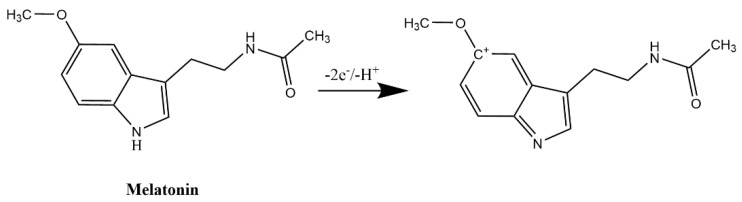
The melatonin oxidation mechanism.

**Figure 5:**
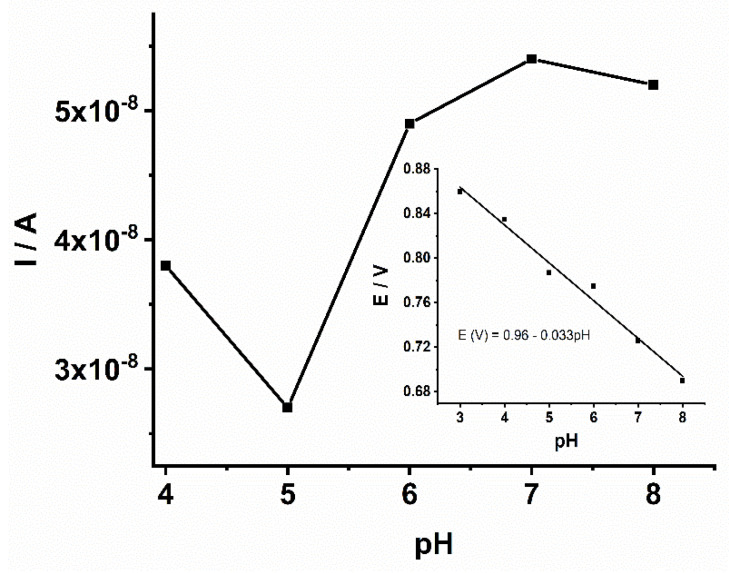
The influence of pH on the response of the SNGCE/AuNP electrochemical sensor in the presence of 10 µM melatonin. Inset: the dependence of the oxidation peak potential on pH.

The electrochemical behavior of melatonin is pH dependent. The selection of the proper supporting electrolyte and buffer is a significant step in electroanalytical studies in light of the fact that the electrolyte composition and pH influence the properties of the solution as well as the electrode/solution interface, modifying the kinetics and thermodynamics of the charge transfer process, and the adsorption at the electrode surface. Therefore, the pH influence on the electrochemical oxidation of melatonin using CA as a detection technique was investigated. It was observed that the oxidation current for an applied working potential of 0.70 V increased for pH values between 5 and 7, while for pH values of 4 and 8, lower currents were recorded (see Figure 5). Moreover, the dependence of the oxidation peak potential on pH was linear (see inset of Figure 5). The slope of the *E_p_* vs. pH dependence suggests that the melatonin oxidation proceeds via the transfer of two electrons accompanied by the transfer of one proton (see Figure 5). Consequently, the melatonin oxidation pathway can be depicted according to Scheme 3 [35]. The selected working potential value of +0.70 V was located after the peak potential value observed for MEL at the SNGCE/AuNP electrode in order to ensure a complete electrochemical oxidation of the analyte. Thus, 0.1 M PBS of pH 7 was selected as the supporting electrolyte in the following analytical studies.

##### Effect of Working Potential

**Figure 6:**
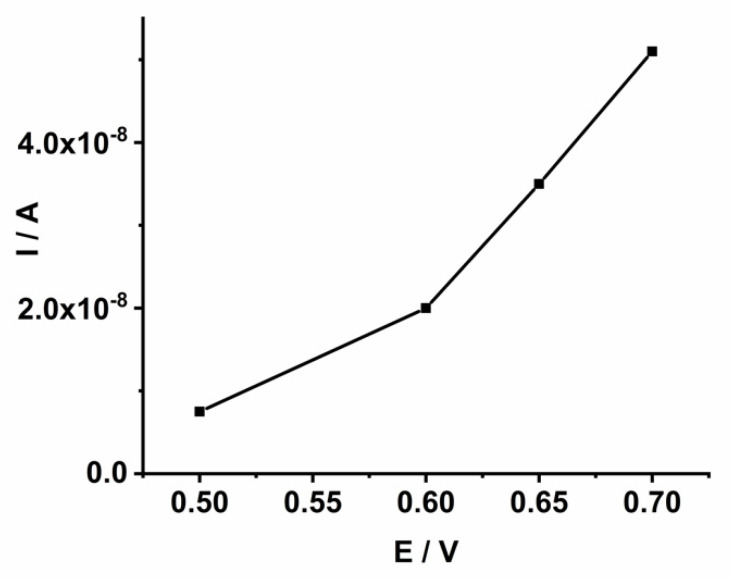
The influence of the working detection potential on the SNGCE/AuNP sensor’s response.

The influence of the working potential in the electrochemical determination of melatonin was also studied. Potential values in the range from 0.50 V to 0.70 V were investigated. All measurements were performed in 0.1 M PBS of pH 7. The experimental results displayed in Figure 6 demonstrate a better analytical signal at higher working potential value. Therefore, the value of 0.70 V was chosen in the following analytical studies.

### 3.3. Analytical Performances of Sensing Materials

**Figure 7:**
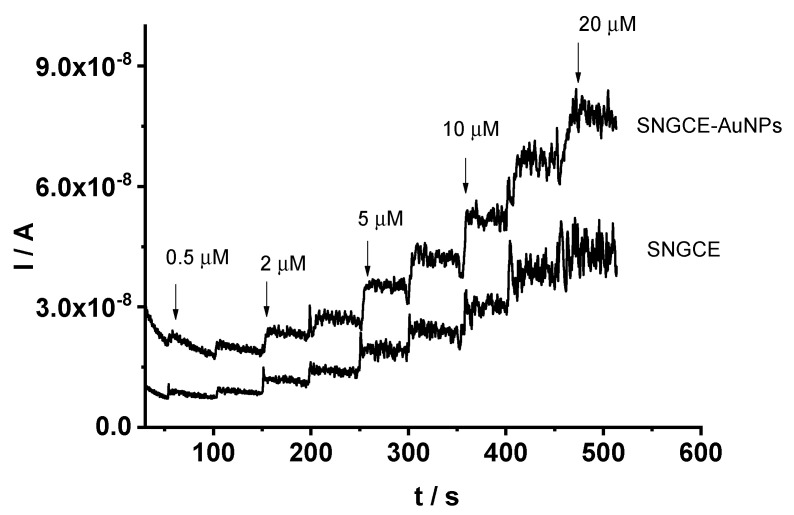
Chronoamperograms recorded at SNGCE and SNGCE/AuNPs in 0.1 M PBS of pH 7 containing different MEL concentrations: 0.5, 1, 2, 3, 5, 7, 10, 15, and 20 µM. E_appl_ = 0.70 V.

The analytical performances of the prepared sensing materials SNGCE and SNGCE/AuNPs toward melatonin determination were investigated by using the chronoamperometry technique. The figures of merit of the analytical performance in terms of linear response range, sensitivity, limits of detection, and quantification were assessed for the SNGCE and SNGCE-AuNP sensing materials. The chronoamperograms recorded at the SNGCE and SNGCE/AuNP sensors in 0.1 M PBS of pH 7 containing different melatonin concentrations are depicted in Figure 7.

The current increased after each melatonin addition, attesting to the fast response of the SNGCE/AuNP sensor. A similar response was observed for SNGCE. At higher melatonin concentrations (i.e., 15 and 20 μM), the analytical signal recorded for SNGCE displayed a quite large noise, which could be due to the fouling of the electrode surface with the oxidation product. In the case of the SNGCE/AuNP sensor, the noise was almost absent, suggesting the good antifouling properties of AuNPs. Consequently, the analytical performance and applications were studied considering the SNGCE and SNGCE/AuNP sensors for a proper comparison. The chronoamperometry technique was used in the assessment of the analytical performance of the proposed SNGCE/AuNP sensor in terms of the limits of detection and quantification, linear response range, repeatability, sensitivity, and response time.

The repeatability, expressed as relative standard deviation (RSD%, *n* = 3) of the slope of the calibration plot, was determined by measuring the analytical signal of melatonin over the concentration range from 0.5 to 20 µM using the same electrodes SNGCE and SNGCE/AuNPs, respectively, three times,. The RSD% values of 5.3% and 1.9% for SNGCE and SNGCE/AuNPs, respectively, were obtained. The limit of detection (LOD) was assessed by using the following criterion: 3*SD/m, where *SD* is the standard deviation of the blank (*n* = 3) and *m* is the slope of the calibration plot. The limit of quantification (LOQ) was assessed by using the criterion 10*SD/m. The SNGCE displayed a linear response toward melatonin oxidation over the concentration range from 0.5 to 20 µM and a detection limit of 100.2 nM, respectively.

**Figure 8:**
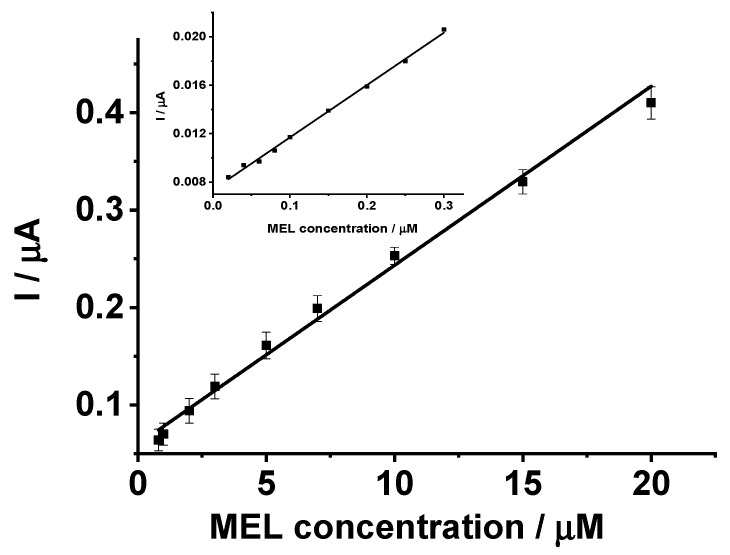
The calibration plot for the SNGCE/AuNP sensor including error bars for melatonin concentrations ranging from 0.5 to 20 μM. Inset: calibration plot for the concentration range from 0.02 to 0.3 μM.

In order to assess the possibility of applying the sensors in the melatonin determination in real samples, the responses of the sensors over lower concentration range below 0.5 µM MEL were investigated. The SNGCE sensor did not show a linear response over the lower concentration range. The SNGCE/AuNP sensor displayed two linear response ranges, the first one from 0.02 to 0.3 µM, and a second one from 0.5 to 20 µM. The calibration plots for the SNGCE/AuNP sensor applied in the melatonin detection including the error bars is depicted in Figure 8. From this figure, it can be observed that the oxidation peak current increased linearly with the melatonin concentration from 0.5 to 20 µM according to the linear regression equation: I_p_ (µA) = 0.05 + 0.019 [MEL] (µM) (r = 0.9956). The sensitivity of the SNGCE/AuNP sensor was 19 µA/mM, computed from the calibration plot. The detection limit was 8.4 nM considering the first linear range comprised between 0.02 and 0.3 µM (inset of Figure 8) with the following linear regression equation I_p_ (nA) = 7.37 + 0.043 [MEL] (nM) (r = 0.9994), and the corresponding sensitivity of 43 nA/µM. The LOQ value of 27.9 nM was obtained for the SNGCE/AuNP sensor considering the lower concentration range. The response time of the SNGCE and SNGCE/AuNP sensors was estimated as the time required to achieve a 95% level from the steady state current when the melatonin concentration was increased from 3 to 5 µM. Consequently, the response time for the SNGCE/AuNP sensor was 5 s. The response time of the SNGCE sensor was assessed similarly and a value of 4 s was obtained. Despite the close response time values, the SNGCE sensor displayed a lower analytical response for melatonin concentrations higher than 10 µM.

It is worth noting that the detection limit obtained for the SNGCE/AuNPs sensor was lower than those previously reported on other sensors for melatonin detection (Table 1). This finding opens the way to potential applications of the SNGCE/AuNP sensor in the analysis of real samples with low melatonin levels.

The analytical performance of the SNGCE/AuNP sensor is comparable to that of other sensors for melatonin reported in the literature both in terms of linear response range and low detection limit (see Table 1). The cost-effective and environmentally friendly sensing materials used in the sensor construction, alongside its simple build and the competitive figures of merit of analytical performance such as linear response range, sensitivity, and detection limit demonstrate the usefulness of the proposed SNGCE/AuNP sensor in the electrochemical sensing of melatonin.

It should be noted that other analytical methodologies such as fast-scan cyclic voltammetry in connection with the carbon fiber microelectrode-based implantable sensor provide real-time monitoring and selective determination of melatonin with a low detection limit value of 24 nM [43]. The use of electrochemical methods such as square wave voltammetry and carbon fiber microelectrodes proves to be a very efficient approach for the selective detection of melatonin both in vitro and in vivo [44]. The monitoring of melatonin in vivo and its quantification in real samples is challenging and the role of many factors such as sampling protocol, sample treatment, and method sensitivity should be considered for a proper comparison of the results [45]. The proposed SNGCE/AuNPs displayed a low detection limit comparable with the previously reported sensors and analytical methodologies, thus this finding underpins the further investigation and prospect for potential applications in real sample analysis.

#### Interference Study

**Figure 9:**
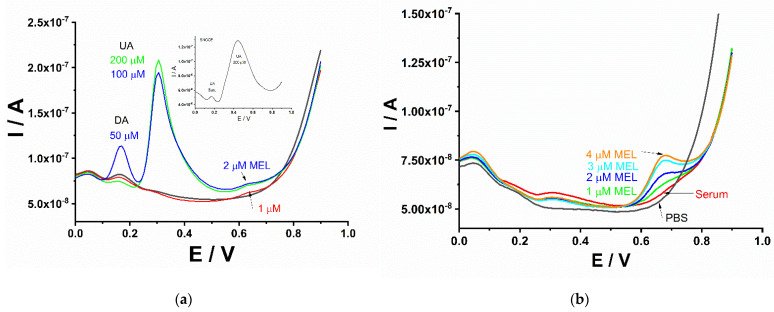
(**a**) DPVs recorded at SNGCE/AuNPs in 0.1 M PBS of pH 7 containing 100 and 200 µM UA, 50 DA, and different MEL concentrations of 1 and 2 µM. Inset: DPV recorded at SNGCE in 0.1 M PBS of pH 7 containing 200 µM UA, 5 µM DA, and 2 µM MEL. (**b**) DPVs recorded at SNGCE/AuNPs in diluted serum sample spiked with various MEL concentrations ranging from 1 to 4 µM.

The major criterion in the assessment of the effectiveness of a sensor is the selectivity. The presence of various interfering species such as uric acid (UA), dopamine (DA), and ascorbic acid (AA) in the human blood can influence the voltammetric response of the developed sensor and can also affect its selectivity. UA is the major interfering specie in the melatonin determination in blood samples because it is present in high concentration levels ranging from ca. 140 to 400 µM. Moreover, the oxidation potential of uric acid is close to that of melatonin and the resolution of the peak potentials of melatonin and uric acid is of paramount importance in light of the real sample analysis. The selectivity study was performed by means of differential pulse voltammetry in order to assess the peak potential separation of various interfering species with respect to melatonin. To this purpose, the differential pulse voltammograms were recorded at the SNGCE/AuNP sensors in the presence of fixed concentrations of interfering species such as 100 and 200 µM UA, 50 µM DA, and at different MEL concentrations of 1 and 2 µM (see Figure 9a). A peak potential separation of 330 mV between the oxidation potentials of MEL and UA was obtained. This peak potential separation ensures the proper determination of MEL in the presence of UA. Similar behavior was obtained in the case of DA. It can be observed that the oxidation peak potential and the peak current response of MEL were not affected by the presence of DA. There was a peak potential separation among MEL and DA of more than 460 mV, which ensures the reliable quantification of MEL in the presence of DA. The presence of AA was revealed by an anodic oxidation peak at ca. 0.07 V, which merged and overlapped with that of DA at ca. 0.18 V after subsequent potential scanning or increase in AA concentration. The small peak at ca. 0.07 V was visible in the blank PBS after the removal of the SNGCE/AuNPs from the test solution containing MEL and the interfering species. However, the overlap of DA and AA peaks did not affect the MEL oxidation potential value. The peak current related to MEL oxidation was not affected by the presence of these interfering species, mainly UA, whose concentration was significantly larger than that of MEL in the biological samples. Therefore, the use of the SNGCE/AuNP sensor in the MEL electroanalysis in real samples with complex compositions such as blood serum samples can be envisaged. In contrast, the use of SNGCE in the interference study revealed a poorer selectivity toward MEL in the presence of UA (inset of Figure 9a). The oxidation wave of UA overlapped that of MEL. Consequently, there is no possibility of quantifying MEL in the presence of UA, despite the small effect of DA contemporary present in the solution.

### 3.4. Recovery Studies

The SNGCE/AuNP sensor was tested in human peripheral blood serum for melatonin determination in order to assess its potential applicability in real sample analysis. To this purpose, the blood serum diluted 20 times with PBS was spiked with 200 nM melatonin and the chronoamperometric response of the sensor was recorded. The obtained concentration value was 198.2 nM and the corresponding recovery was 99.1%. This result attests to the good accuracy of the sensor and its applicability to real samples with a complex composition. The DPVs were also recorded in the serum sample after 20 times dilution with PBS and followed by spiking with various MEL concentrations (see Figure 9b). The DPV traces demonstrate that the SNGCE/AuNP sensor can discriminate MEL in the presence of UA, this being ascribed to the anodic wave located at ca. 0.3 V, similar to the data displayed in Figure 9a. There was a small shift in MEL oxidation potential from 0.64 to 0.68 V compared to the interference data in synthetic samples from Figure 9a, which could be due to the matrix effect of the real sample. However, this potential shift of ca. 40 mV was relatively small. Therefore, the proposed SNGCE/AuNPs displayed overall good analytical performance successfully demonstrated in both synthetic and real sample analysis with complex chemical composition.

## 4. Conclusions

The preparation and characterization of various sensing materials based on Sonogel-Carbon and Au nanoparticles were carried out. The sonochemical synthesis of AuNPs provided a narrow size distribution of the obtained nanoparticles with a small average size diameter less than 15 nm. The prepared materials displayed good electrochemical properties tested by means of cyclic voltammetry in the presence of a redox probe. The use of AuNPs improved electron transfer capability by ca. 25% and enhanced analytical sensitivity compared to SNGCE. The best analytical performance toward melatonin was obtained for the SNGCE/AuNP sensor with a wide linear response range, low detection limit, and good accuracy in real sample analysis. The SNGCE/AuNP sensor displayed two linear response ranges, the first one from 0.02 to 0.3 µM, and a second one from 0.5 to 20 µM, respectively. A very low detection limit of 8.4 nM, considering the lower concentration range, was obtained. The selective detection of melatonin in the presence of interfering species such as uric acid, ascorbic acid, and dopamine was successfully achieved. The SNGCE/AuNP sensor displayed good anti-interference capability in human peripheral blood serum sample analysis and overall analytical performance comparable with that of other sensors reported in the literature. The matrix influence study in real samples revealed a recovery value of 99.1%, demonstrating the good accuracy of the proposed sensor. The affordable cost of the sensing material, the eco-friendly preparation procedure, and the simple construction of the SNGCE/AuNP sensor, alongside the good analytical performance demonstrate the usefulness of the proposed sensing material and analytical strategy for melatonin determination. The obtained results point out the potential applications of the proposed sensor in the electroanalysis of samples with complex matrix composition for melatonin detection. The proposed methodology could be further extended to the study of other metal nanoparticles as sensing elements together with the Sonogel-Carbon electrode material in the development of electrochemical sensors for relevant biologically active compounds such as neurotransmitters and antioxidants.

## Figures and Tables

**Table 1:** Comparison of the analytical performance of SNGCE/AuNPs with other electrochemical sensors.

Sensor	Linear Response Range (μM)	Detection Limit (nM)	Ref.
SnO_2_-Co_3_O_4_@rGO/IL/CPE	0.02–6	4.1	[17]
3.4′AAZCPE	0.3–10	56	[18]
WP	0.8–100	32.5	[19]
B-RGO	2.3–2000	700	[20]
GBGC	0.0028–12	19	[22]
MIP	10–80	140	[37]
Au-MoS_2_/GCE	0.033–10	15.7	[38]
Gr-Fe_3_O_4_/CPE	0.02–5.80	8.4	[39]
GPH-CSPE	1–300	870	[40]
Gr-AV	10–100	490	[41]
GCE/MagNPs/Cdots	0.05–13.50	4.4	[42]
FSCV	0.05–10	24	[43]
CFEs	0.1–5	38.1	[44]
SNGCE	0.5–20	100.2	This work
SNGCE-AuNPs	0.02–0.3	8.4	This work

SnO_2_-Co_3_O_4_@rGO/IL/CPE-ionic liquid carbon paste electrode modified with reduced graphene oxides decorated with SnO_2_-Co3O4 NPs; 3,4’AAZCPE is an electrode composed of ZnO nanorods carbon paste electrode (ZCPE) modified with 3-(4′-amino-3′-hydroxy-biphenyl-4-yl)-acrylic acid; WP—waterproof paper; B-RGO-paper electrode realized through the dispersion of B_2_O_3_ in GO; MIP—molecular imprinted polymer; Gr-Fe_3_O_4_/CPE-graphene decorated with Fe_3_O_4_ magnetic NPs on a C paste electrode; GPH-CSPE—graphene-carbon screen-printed electrode; Gr-AV—graphite/automotive varnish ink; GBGC—carbon black suspended in dimethylformamide on glassy carbon electrode; GCE/MagNPs/Cdots—GCE coated with Fe_3_O_4_ nanoparticles and carbon quantum dots (MagNPs/Cdots); FSCV—fast scan cyclic voltammetry. CFEs—carbon fiber microelectrodes.

## Data Availability

Data are contained within the article.

## References

[1] Farhadi N., Gharghani M., Farhadi Z. (2016). Effects of long-term light, darkness and oral administration of melatonin on serum levels of melatonin. Biomed. J..

[2] Pfeffer M., Korf H.W., Wicht H. (2018). Synchronizing effects of melatonin on diurnal and circadian rhythms. Gen. Comp. Endocrinol..

[3] Alzoubi K.H., Mayyas F.A., Mahafzah R., Khabour O.F. (2018). Melatonin prevents memory impairment induced by high-fat diet: Role of oxidative stress. Behav. Brain Res..

[4] Esteban-Zubero E., López-Pingarrón L., Alatorre-Jiménez M.A., Ochoa-Moneo P., Buisac-Ramón C., Rivas-Jiménez M., Castán-Ruiz S., Antoñanzas-Lombarte A., Tan D.X., García J.J. (2017). Melatonin’s role as a co-adjuvant treatment in colonic diseases: A review. Life Sci..

[5] Xie Z., Chen F., Li W.A., Geng X., Li C., Meng X., Feng Y., Liu W., Yu F. (2017). A review of sleep disorders and melatonin. Neurol. Res..

[6] Srinivasan V., Brzezinski A., Perumal S.R.P., Spence D.W., Cardinali D.P., Brown G.M. (2011). Melatonin agonists in primary insomnia and depression-associated insomnia: Are they superior to sedative-hypnotics?. Prog. Neuro-Psychopharmacol. Biol. Psychiatry.

[7] Meng T., Zheng Z.H., Liu T.T., Lin L. (2012). Contralateral retinal dopamine decrease and melatonin increase in progression of hemiparkinsonium rat. Neurochem. Res..

[8] Escrivá L., Manyes L., Barberà M., Martínez-Torres D., Meca G. (2016). Determination of melatonin in Acyrthosiphon pisum aphids by liquid chromatography-tandem mass spectrometry. J. Insect. Physiol..

[9] Zhao H., Wang Y., Yuan B., Liu S., Man S., Xu H., Lu X. (2016). A novel LC-MS/MS assay for the simultaneous determination of melatonin and its two major metabolites, 6-hydroxymelatonin and 6-sulfatoxymelatonin in dog plasma: Application to a pharmacokinetic study. J. Pharm. Biomed. Anal..

[10] Martins L.G., Khalil N.M., Mainardes R.M. (2017). Application of a validated HPLC-PDA method for the determination of melatonin content and its release from poly(lactic acid) nanoparticles. J. Pharm. Anal..

[11] Lu J., Lau C., Lee M.K., Kai M. (2002). Simple and convenient chemiluminescence method for the determination of melatonin. Anal. Chim. Acta.

[12] Oladi E., Mohamadi M., Shamspur T., Mostafavi A. (2014). Spectrofluorimetric determination of melatonin in kernels of four different Pistacia varieties after ultrasound-assisted solid-liquid extraction. Spectrochim. Acta Part A Mol. Biomol. Spectrosc..

[13] Pola M.L., Algarra M., Becerra A., Hernandez M. (2000). Cyclodextrin enhanced spectrofluorimetric determination of melatonin in pharmaceuticals and urine. Anal. Lett..

[14] Li Y., Cassone V.M. (2015). A simple, specific high-throughput enzyme-linked immunosorbent assay (ELISA) for quantitative determination of melatonin in cell culture medium. Int. Immunopharmacol..

[15] Patel B.A. (2008). Continuous amperometric detection of co-released serotonin and melatonin from the mucosa in the ileum. Analyst.

[16] Manikandan P.N., Dharuman V. (2017). Electrochemical simultaneous sensing of melatonin, dopamine and acetaminophen at platinum doped and decorated alpha iron oxide. Electroanalysis.

[17] Zeinali H., Bagheri H., Monsef-Khoshhesab Z., Khoshsafar H., Hajian A. (2017). Nanomolar simultaneous determination of tryptophan and melatonin by a new ionic liquid carbon paste electrode modified with SnO2-Co3O4@rGO nanocomposites. Mater. Sci. Eng. C.

[18] Molaakbari E., Mostafavi A., Beitollahi H. (2015). Simultaneous electrochemical determination of dopamine, melatonin, methionine and caffeine. Sens. Actuators B Chem..

[19] Camargo J.R., Andreotti I.A.A., Kalinke C., Henrique J.M., Bonacin J.A., Janegitz B.C. (2020). Waterproof paper as a new substrate to construct a disposable sensor for the electrochemical determination of paracetamol and melatonin. Talanta.

[20] Topcu E., Kıranşan K.D. (2020). Electrochemical simultaneous sensing of melatonin and ascorbic acid at a novel flexible B-RGO composite paper electrode. Diam. Relat. Mater..

[21] Duan D., Ding Y., Li L., Ma G. (2020). Rapid quantitative detection of melatonin by electrochemical sensor based on carbon nanofibers embedded with FeCo alloy nanoparticles. J. Electroanal. Chem..

[22] Smajdor J., Piech R., Pięk M., Paczosa-Bator B. (2017). Carbon black as a glassy carbon electrode modifier for high sensitive melatonin determination. J. Electroanal. Chem..

[23] Kumar N., Goyal R.N. (2016). Nanopalladium grained polymer nanocomposite based sensor for the sensitive determination of melatonin. Electrochim. Acta.

[24] Qu W., Wang F., Hu S., Cui D. (2005). Electrocatalytic properties and voltammetric determination of melatonin at a nanostructured film electrode. Microchim. Acta.

[25] García-Guzmán J.J., Hernández-Artiga M.P., Palacios-Ponce de León L., Bellido-Milla D. (2015). Selective methods for polyphenols and sulphur dioxide determination in wines. Food Chem..

[26] Naranjo-Rodriguez I., Domínguez M., Hernández-Artiga M.P., Bellido-Milla D., Hidalgo-Hidalgo de Cisneros J.L. (2008). A third-generation hydrogen peroxide biosensor based on Horseradish Peroxidase (HRP) enzyme immobilized in a Nafion-Sonogel-Carbon composite. Electrochim. Acta.

[27] Attar A., Cubillana-Aguilera L., Naranjo-Rodriguez I., Hidalgo-Hidalgo de Cisneros J.L., Palacios-Santander J.M., Amine A. (2015). Amperometric inhibition biosensors based on horseradish peroxidase and gold sononanoparticles immobilized onto different electrodes for cyanide measurements. Bioelectrochemistry.

[28] Ajaero C., Abdelrahim M.Y.M., Palacios-Santander J.M., Almoraima Gil M.A., Naranjo-Rodríguez I., Hidalgo-Hidalgo de Cisneros J.L., Cubillana-Aguilera L. (2012). Comparative study of the electrocatalytic activity of different types of gold nanoparticles using Sonogel-Carbon material as supporting electrode. Sens. Actuators B Chem..

[29] Bellido-Milla D., Cubillana-Aguilera L., El Kaoutit M., Hernández-Artiga M.P., Hidalgo-Hidalgo de Cisneros J.L., Naranjo-Rodríguez I., Palacios-Santander J.M. (2013). Recent advances in graphite powder-based electrodes. Anal. Bioanal. Chem..

[30] Rahmati R., Hemmati A., Mohammadi R., Hatamie A., Tamjid E., Simchi A. (2020). Sensitive voltammetric detection of melatonin in pharmaceutical products by highly conductive porous graphene-gold composites. ACS Sustain. Chem. Eng..

[31] Bottari D., Pigani L., Zanardi C., Terzi F., Paţurcă S.V., Grigorescu S.D., Matei C., Lete C., Lupu S. (2019). Electrochemical sensing of caffeic acid using gold nanoparticles embedded in poly(3,4-ethylenedioxythiophene) layer by sinusoidal voltage. Chemosensors.

[32] Cubillana-Aguilera L.M., Palacios-Santander J.M., Naranjo-Rodriguez I., Hidalgo-Hidalgo de Cisneros J.L. (2006). Study of the influence of the graphite powder particle size on the structure of the Sonogel-Carbon materials. J. Sol-Gel Sci. Technol..

[33] Cubillana-Aguilera L.M., Franco-Romano M., Gil M.L.A., Naranjo-Rodriguez I., Hidalgo-Hidalgo de Cisneros J.L., Palacios-Santander J.M. (2011). New, fast and green procedure for the synthesis of gold nanoparticles based on sonocatalysis. Ultrason. Sonochem..

[34] Laviron E. (1974). Adsorption, autoinhibition and autocatalysis in polarography and in linear potential sweep voltammetry. J. Electroanal. Chem..

[35] Levent A. (2012). Electrochemical determination of melatonin hormone using a boron-doped diamond electrode. Diam. Relat. Mater..

[36] Alpar N., Pınar P.T., Yardım Y., Şentürk Z. (2017). Voltammetric method for the simultaneous determination of melatonin and pyridoxine in dietary supplements using a cathodically pretreated boron-doped diamond electrode. Electroanalysis.

[37] Zembrzuska D., Kalecki J., Cieplak M., Lisowski W., Borowicz P., Noworyta K., Sharma P.S. (2019). Electrochemically initiated co-polymerization of monomers of different oxidation potentials for molecular imprinting of electroactive analyte. Sens. Actuators B Chem..

[38] Selvam S.P., Hansa M., Yun K. (2020). Simultaneous differential pulse voltammetric detection of uric acid and melatonin based on a self-assembled Au nanoparticle–MoS2 nanoflake sensing platform. Sens. Actuators B Chem..

[39] Bagheri H., Afkhami A., Hashemi P., Ghanei M. (2015). Simultaneous and sensitive determination of melatonin and dopamine with Fe3O4 nanoparticle-decorated reduced graphene oxide modified electrode. RSC Adv..

[40] Apetrei I.M., Apetrei C. (2016). Voltammetric determination of melatonin using a graphene-based sensor in pharmaceutical products. Int. J. Nanomed..

[41] Freitas R.C., Orzari L.O., Ferreira L.M.C., Paixão T.R.L.C., Coltro W.K.T., Vicentini F.C., Janegitz B.C. (2021). Electrochemical determination of melatonin using disposable self-adhesive inked paper electrode. J. Electroanal. Chem..

[42] Cincotto F.H., Carvalho D.A.S., Canevari T.C., Toma H.E., Fatibello-Filho O., Moraes F.C. (2018). A nano-magnetic electrochemical sensor for the determination of mood disorder related substances. RSC Adv..

[43] Austin L., Hensley A.L., Colley A.R., Ross A.E. (2018). Real-time detection of melatonin using fast-scan cyclic voltammetry. Anal. Chem..

[44] Castagnola E., Woeppel K., Golabchi A., McGuier M., Chodapaneedi N., Metro J., Mitch Taylor I., Tracy Cui X. (2020). Electrochemical detection of exogenously administered melatonin in the brain. Analyst.

[45] Rzepka-Migut B., Paprocka J. (2020). Melatonin-measurement methods and the factors modifying the results. A systematic review of the literature. Int. J. Environ. Res. Public Health.

